# An investigation into managerial support for the psychological wellbeing of national health service doctors during the Covid‐19 pandemic: A cross sectional study

**DOI:** 10.1002/hpm.3564

**Published:** 2022-08-26

**Authors:** Salman Ahmed Abdul Jabbar, Carol Marshall

**Affiliations:** ^1^ Stirling Management School University of Stirling Stirling, Scotland UK

**Keywords:** Covid‐19, doctors, healthcare management, managerial support, psychological wellbeing

## Abstract

**Aim:**

This study investigates the psychological wellbeing of United Kingdom National Health Service doctors during the Covid‐19 pandemic and evaluates how they have been supported managerially.

**Method:**

A mixed‐method sequential study design of online surveys and semi‐structured interviews was employed between July‐August 2020, with a response rate of 273/300 and 4/4 respectively. The Warwick‐Edinburgh Mental Wellbeing Scale (WEMWBS) and Health and Safety Executive Management Standards (HSE MS) were used as measuring tools. The Jobs Demands Resource (JD‐R) model and its relation to psychological wellbeing was determined. Survey findings informed semi‐structured interviews, coded using thematic analysis.

**Results:**

Overall mean WEMWBS, 43.2 (SD = 9.44), was low as was mean managerial support, 2.38 (SD = 0.78). Overall mean clinical demand score was high (2.6 on reverse scale). First year female trainee respondents from frontline specialties were found to have low psychological wellbeing scores. Key correlations were found between high managerial support, low clinical demands and low psychological wellbeing (*r* > 0.6). Core themes emerged: (1) breakdown of leadership, (2) vulnerability of wellbeing without support, (3) suboptimal navigation through change and (4) poor physical and human resource management.

**Conclusion:**

Maintaining the psychological wellbeing of doctors requires physical and psychological resources to meet clinical demands and the enhancement of fundamental managerial principles of control, communication, change management and leadership through adversity.

## Highlights

- Frontline doctors face a surge in work demands during the Covid‐19 pandemic.
- Doctors' psychological wellbeing vulnerable without effective national health service (NHS) management.
- Safety, communication and resource to meet demands for NHS doctors is paramount.
- Novel use of the Jobs Demands‐Resource Model for NHS management during Covid‐19.

## 1 BACKGROUND

The Coronavirus Disease 2019 (Covid‐19) pandemic is an unprecedented crisis with over 135 million cases and over 2.5 million deaths worldwide, posing extreme public health concerns.^1^ In particular, the United Kingdom has experienced high morbidity and mortality rates placing immense pressure on the NHS and its workforce.^2^

### 1.1 Psychological wellbeing

During the current Covid‐19 crisis, clinicians are having to make difficult decisions and are having to balance their personal needs with those of their patients.^3^ Early studies, including a recent systematic review and meta‐analysis on Covid‐19, have shown rising psychological strain amongst doctors influenced by increased clinical demand, staff shortages and lack of Personal Protective Equipment (PPE).^4^ With rapid progression of the virus since its outbreak and lack of preparedness amongst healthcare organisations, it is clear that there are early gaps in the evidence‐based evaluation of Covid‐19‐related distress amongst medical healthcare workers.^5^, ^6^

### 1.2 Managerial support

Times of crises are a period of destabilisation for organisations, and managers must have the ability to make quick decisions, provide support and give direction.^7^ Studies have shown that organisational measures during the early pandemic to address workplace hygiene standards and concerns result in less psychiatric symptoms.^8^ Stoller^9^ determined key competencies and strategies which are necessary for leadership to display during a crisis. These include proactivity, robust governance structures, quick implementation of initiatives, communication and being both realistic and optimistic.^9^ Additionally, the system capacity to absorb disturbance, carry out change and continue to retain the same functionalities and structure measures the resilience of an organisation and its management.^10^

There are multiple theoretical frameworks which underpin the impact of managerial support on occupational psychological wellbeing; For example, the Jobs Demands‐Resources (JD‐R) Model used by organisations to improve employee health and motivation and the Health & Safety Executive Management Standards Indicator Tool (HSE‐MS IT), which assesses work‐related stress risks at an organizational level.^11^, ^12^, ^13^

The JD‐R Model involves two factors: Job demands and Job resources which can be used to predict negative work outcomes.^14^ The model proposes two underlying psychological processes. The first is that job demands utilise and reduce resources both physical and psychological, and this in effect results in exhaustion. This loss of energy has been evidenced as negatively affecting employee wellbeing.^15^ The second process suggests that resources give a sense of reward, which can increase motivation and improve health and wellbeing.^16^

The United Kingdom Management Standards were designed by the Health and Safety Executive (HSE) in order to reduce the level of work‐related stress and is based on six categories of the psychosocial work environment.^17^ These dimensions include demands, control, support, role, change and relationships. Through these standards an HSE‐MS IT was created, which is a questionnaire designed to capture an organisations performance against the six dimensions. This can facilitate identification of work characteristics such as demands, resources, support and how, if these are not managed appropriately, they can lead to poor employee wellbeing.^18^

Whilst studies have expanded on the JD‐R framework to model how job resources in terms of management support for safety can impact the relationship with demands and hazardous work events, to the best of our knowledge, there is no study which has examined this in the context of the Covid‐19 pandemic.^19^ Similarly, the assessment of management policies using the HSE management standards has, to the best of our knowledge, not been described in relation to the wellbeing of doctors during the Covid‐19 pandemic.

### 1.3 Study purpose

It is unsurprising that, within the NHS, there has been a call for action to address the growing concern of psychological wellbeing amongst vulnerable groups, which includes healthcare and frontline workers, and prioritise research to determine strategies which can be implemented with immediate effect.^20^, ^21^ If psychological wellbeing is not understood from a managerial perspective and doctors remain unsupported, the NHS could see a breakdown in the performance of their workers due to burnout and distress; Therefore, research within medical management is imperative.^22^ Studies have investigated the relationship between wellbeing of employees in the healthcare setting and have shown that creating a sense of community through leadership can have a positive influence on wellbeing in the workplace.^23^ Equally, other studies have highlighted that perception of leadership style in the health service can have a substantial influence on employee wellbeing.^24^ The question arises as to whether managers are taking the necessary steps to accommodate the strain on the NHS and whether they are successfully implementing adequate policies and strategies to ensure the psychological wellbeing of their healthcare employees is maintained.

The aim of this study was to determine the psychological wellbeing of NHS doctors during the Covid‐19 pandemic and evaluate the perception of clinical demand and the effectiveness of managerial support. An additional aim was to determine relationships between managerial interventions, clinical demand and psychological wellbeing. The impact of managerial practice and interventions on the psychological wellbeing of NHS doctors was also evaluated with the intention to provide recommendations to inform NHS management policy and practice for future high‐pressure crises. Differences in terms of NHS England and Scotland were evaluated given that both health services have independent governing bodies, policies and funding.

## 2 METHODS

### 2.1 Study design

A mixed‐methods approach was chosen for the purpose of this study with data collection between July‐August 2020. Although there are various mixed‐method designs described in the literature, a well‐recognised approach chosen for this study is the sequential explanatory design, where quantitative data is analysed initially, followed by a second phase of qualitative data.^25^, ^26^ The rationale for this approach is that quantitative data provides a general understanding of doctors' wellbeing and the influence of managerial support, whilst qualitative data, through individual semi‐structured interviews, refines and explores these investigative areas in more depth. Essentially, the mixed‐method approach evaluates the perception of managerial support, clinical demand and its influence on the multifaceted topic of psychological wellbeing, but also generates new ideas and inferences to fully explain its complexities. Additionally, researchers advocating the use of a mixed‐methods approach explain that its use is superior in understanding complex contextual situations in real‐world environments compared to traditional quantitative or qualitative approaches alone.^27^, ^28^

### 2.2 Research participants/sampling

The UK NHS is divided into primary and secondary care. Primary care is the first point of contact for patients seeking healthcare in the community delivered by General Practitioners (GPs) and secondary care is delivered in the hospital setting. Doctors progress through training from basic foundation year (FY) 1 and 2 and progress to more senior roles known as core trainees (CT), general practice specialty trainees (GPST) or middle grades (Registrars) who are the most senior trainees. Once training is complete the most senior medical practitioner is known as a consultant. The UK NHS employs over 300,000 doctors. This study aimed to survey 300 doctors including 4 interviewees. Although this sample size is only a fraction of the total number of doctors in the health service this was felt to be achievable within the study period with the aim to maximise representation of the NHS within both primary and secondary care, whilst also investigating a variety of departments and grades of doctors. Although there were 273 of 300 survey respondents, all survey responses for all study domains were included within the analysis to avoid selection bias.

Participants were invited through professional networks including NHS mailing lists from a variety of hospitals in Scotland and England, describing the nature of study and offering voluntary participation. Convenience sampling methods were used, and participation was voluntary and anonymous. Ethical approval was granted by the institution's ethical review committee (Approval code: NHS, Invasive or Clinical Research (NICR) 19/20–092). Electronic informed consent was obtained prior to surveys and interviews.

The principle inclusion criteria of the respondents were: 1. English speaking doctors of the NHS residing in the United Kingdom; 2. Qualified and practicing doctors from the level of foundation to consultant level; 3. Doctors who are actively working or have worked during the Covid‐19 pandemic; 4. Doctors registered with the General Medical Council with a license to practice. The aim was to gain respondents from different specialties and different NHS boards/trusts nationally to ensure accurate representation of the organizational efforts during the pandemic.

### 2.3 Instruments

#### 2.3.1 Surveys

The questionnaire was created as an internet‐based survey, via the platform OnlineSurveys, and consisted of four parts. The survey was emailed to doctors with a response rate of 273/300. The first part of the survey established general demographics including age, sex, clinical department and NHS Region. The second part explored psychological wellbeing and utilised the 14‐item Warwick‐Edinburgh Mental Wellbeing Scale (WEMWBS) which involves aspects of emotional affect, cognitive evaluation and psychological functioning. Higher scoring represents better mental wellbeing. The WEMWBS has been evidenced as a well‐received scale by survey participants and was developed from an NHS based initiative in 2005. The scale's internal consistency and reliability is well evidenced.^29^ The third and fourth parts of the questionnaire involved a series of questions focussing on clinical demands and managerial support. 11 items measuring demands and management support were adapted from the HSE‐MS IT. The HSE MS, being a UK based guideline, is relatable and the measuring tool includes areas of work demand and perceived management support, relevant to the research aims.^17^ Three items regarding safety were adapted from Turner et al.^19^ and the questions chosen were applicable to managerial support for safety and were representative of worker experiences in the clinical environment.^19^

#### 2.3.2 Interviews

Following the survey completion and analysis of data, 4 interviewees were invited via email through convenience sampling with a complete response rate. Interviewees were chosen with the aim to create a diverse group according to the independent variables of age, gender, grade and department. Semi‐structured interviews of 4 doctors were conducted and designed to probe questions related to wellbeing, clinical demand and perception of managerial support. Flexibility was allowed for respondents to discuss aspects which are important to them following their line of thought. Interviews were conducted on an internet platform (Microsoft Teams) and data transcribed and analysed with thematic coding. Interviews lasted approximately 45 min.

The approach to thematic coding was to code the insights gained from transcripts using the software NVivo. Following identification, these codes were analysed to identify commonality using an inductive approach. The purpose of this inductive approach was to identify similar, significant or dominant themes inherent in the data, the benefits of which are to broaden and increase clarity of findings by avoiding the restraints imposed by a structured deductive approach.^30^ Diagrams were used to focus on these emerging themes and help identify relationships with a view to form more focussed themes. These themes were then incorporated into the findings and discussion to enhance the understanding of the study's concepts.

### 2.4 Data analysis

In this study the online survey results guided development of the semi‐structured interviews and findings of both were integrated during overall analysis.

Qualitative data was analysed using IBM SPSS 24.0 (IBM, Armonk, NY, USA). Descriptive measures were established for each independent variable and comparisons made to UK population standards where possible. Independent *t*‐test and Analysis of Variance (ANOVA) testing was utilised to assess variances between demographics. Pearson's correlation and linear regression analyses were used to assess relationships between psychological wellbeing, clinical demand and managerial support. The value *p* < 0.05 was considered statistically significant.

Qualitative semi‐structured interviews were audio recorded through Microsoft Teams. Transcripts were coded using NVivo software (QSR International, Melbourne, Australia) which were then analysed to generate subthemes and core themes.

## 3 RESULTS

### 3.1 Survey results

273 participants completed the online survey. 261 respondents declared their age giving a mean age of 36.4 (Table 1). There was a higher male to female ratio, 145 (53.1%): 128 (46.9%). The mode for age distribution was 25 and there was a significant difference between male age (Mean = 39) and female age (Mean = 33.5): *t* = 3.987, (*df* = 252.2), *p* < 0.05.

**TABLE 1:** Demographic and descriptive data

Variable	*N*	Mean	SD
Study population	273		
Age	261	36.40	11.67
Gender			
Male	145 (53.1%)		
Female	128 (46.9%)		
Department			
Medicine	92 (33.7%)		
Surgery	86 (31.5%)		
A&E	32 (11.7%)		
GP	53 (19.4%)		
Other	10 (3.7%)		
Grade			
FY1	44 (16.1%)		
FY2/SHO/CT/GPST^a^	56 (20.5%)		
Registrar	60 (22%)		
Consultant	70 (25.6%)		
GP	43 (15.8%)		
Wellbeing score	273	43.24	9.44
Clinical demand	273	2.60	0.84
Managerial support	273	2.38	0.78
NHS scotland	131 (48%)		
Age	130	34.92	11.10
Wellbeing score	131	40.90	8.61
Clinical demand	131	2.34	0.72
Managerial support	131	2.18	0.62
NHS England	142 (52%)		
Age	131	37.94	12.07
Wellbeing score	142	45.39	9.68
Clinical demand	142	2.86	0.88
Managerial support	142	2.57	0.85

^a^ Abbreviations: CT, Core Trainee; FY, Foundation Year; GPST, General Practice Specialty Trainee; SHO, Senior House Officer.

Respondents were from primary and secondary care departments including medicine, surgery, A&E (Accident and Emergency i.e. Emergency Medicine) and General Practice (Table 1). Medicine and surgery included all subspecialties; however, the specific specialty was not noted in the survey questionnaire. Secondary care doctors had the highest representation, in particular medicine and surgery with 92 (33.7%) and 86 (31.5%) responses respectively. Representation of the primary care sector came from 53 (19.4%) general practitioners.

There was a marginally higher number of responses from NHS England (52%) in comparison to NHS Scotland (48%). Regarding the grade/level of doctors, there was an even distribution (Table 1).

#### 3.1.1 Wellbeing

**FIGURE 1:**
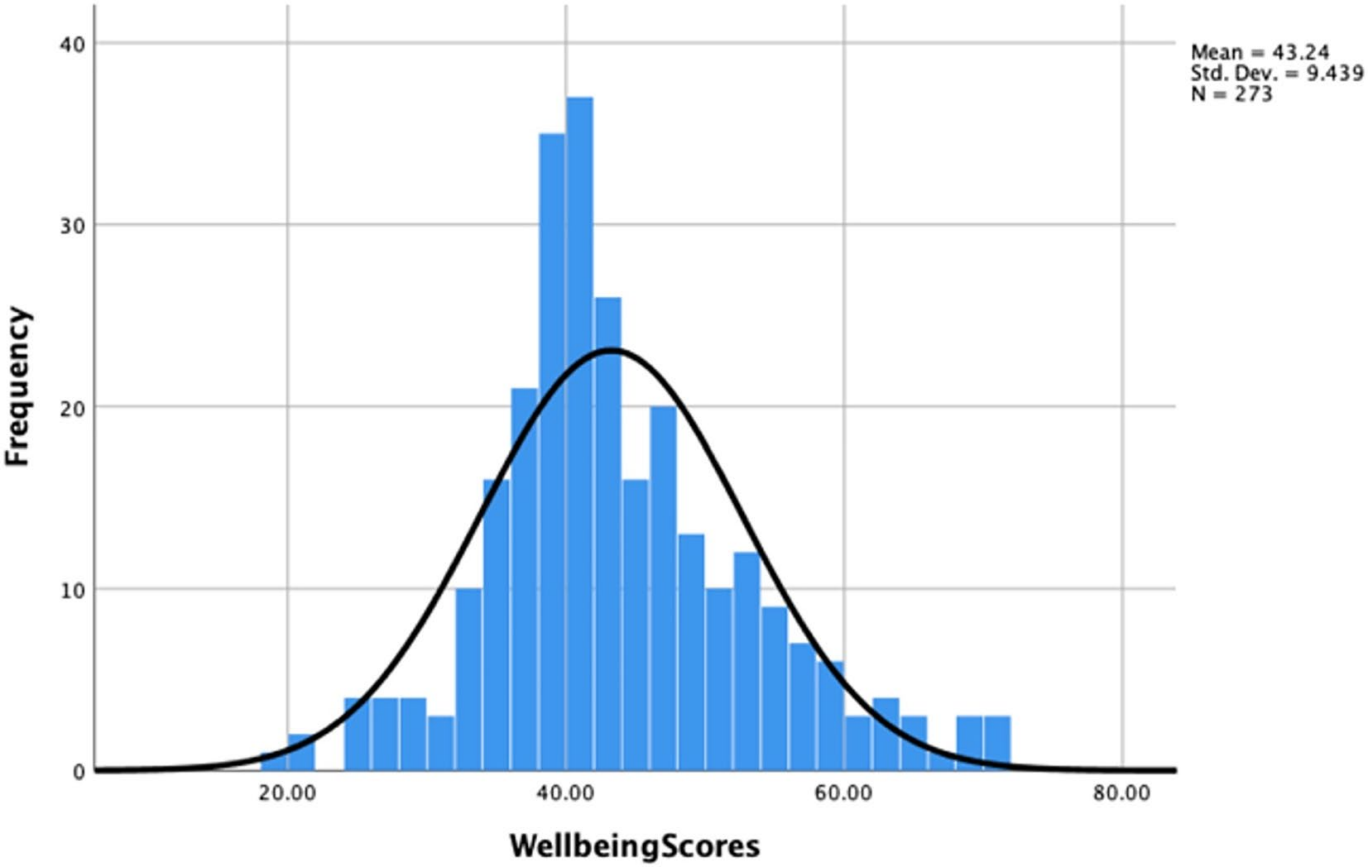
WEMWBS Scores for Study Population

**FIGURE 2:**
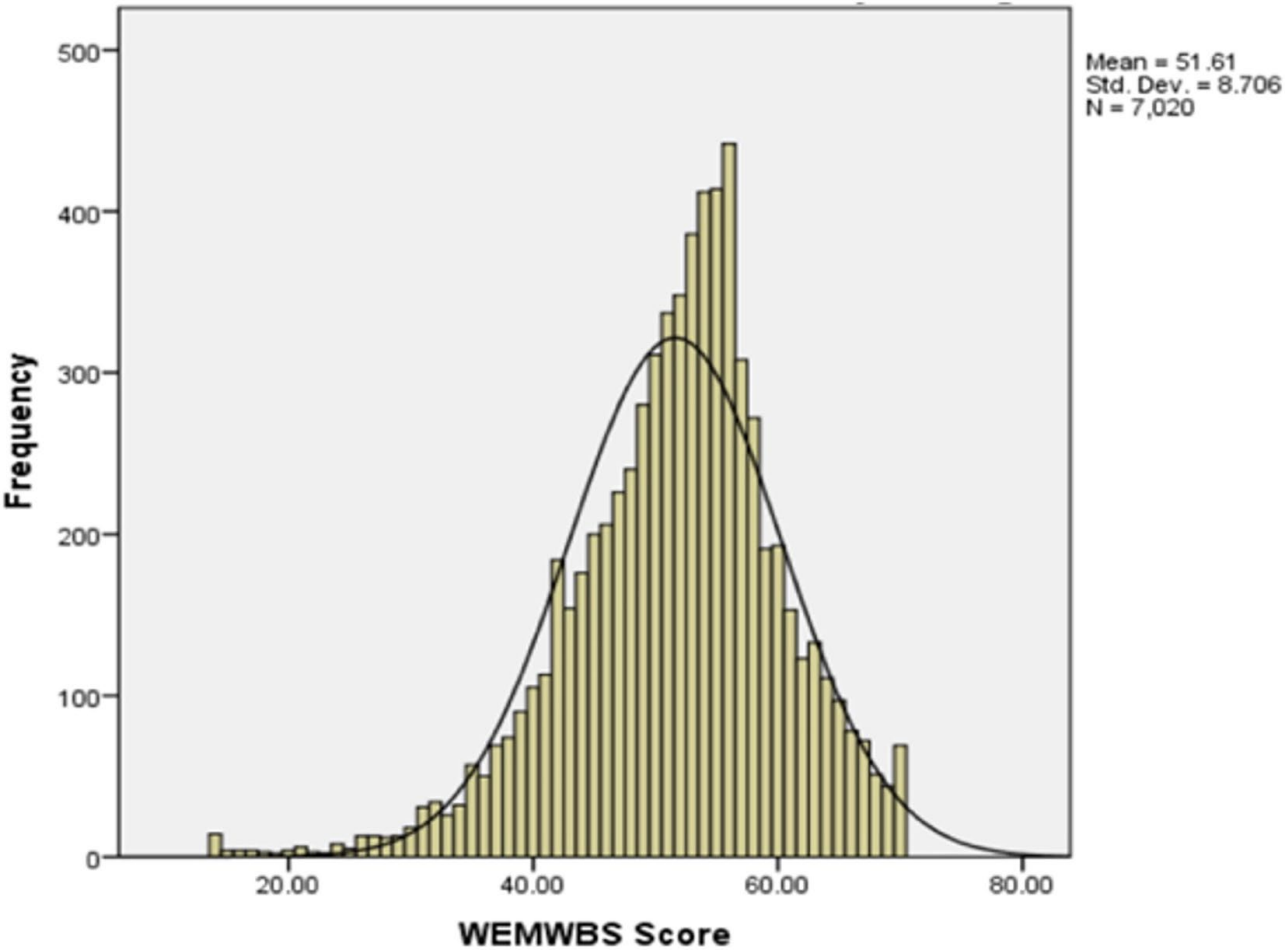
WEMWBS Scores—Health Survey for England 2011^40^

Overall mean WEMWBS score was 43.2 (Standard Deviation (SD) of 9.44) for the study population (Tables 1 and 2) and was lower in comparison to a population mean wellbeing score of 51.6 (SD = 8.7) according to Health Survey for England 2011, *t* = 75.6, (df = 272), *p* < 0.05 (Figures 1 and 2, Table 2).^31^

**TABLE 2:** Independent *T*‐test and Analysis of Variance (ANOVA) analyses of wellbeing score

WEMWBS score				
Study group (*n* = 273)	Population (*n* = 7020)		Independent (i) *t*‐test	
Mean (SD)	Mean (SD)	*t*	df	*p*
43.24 (9.44)	51.60 (8.70)	75.60	272.0	<0.05

NHS scotland (*n* = 131)	NHS England (*n* = 142)		*i t*‐test	
Mean (SD)	Mean (SD)	t	df	*p*
40.90 (8.61)	45.39 (9.68)	−4.05	270.6	<0.05

Male (*n* = 145)	Female (*n* = 128)		*i t*‐test	
Mean (SD)	Mean (SD)	*t*	df	*p*
45.30 (10.48)	40.90 (7.48)	4.04	260.1	<0.05

Medicine (*n* = 92)	Surgery (*n* = 86)		ANOVA	
Mean	Mean	Mean difference	SE	*p*
2.33^a^	2.83	−4.24	1.396	<0.05

^a^ Range: 1 (Low)—5 (High).

WEMWBS score was lower for NHS Scotland respondents (Mean = 40.91) in comparison to NHS England (Mean = 45.39), *t* = −4.05, (df = 270.6), *p* < 0.05 (Table 2). There was also a statistically significant difference between male WEMWBS score (Mean = 45.3) and female WEMWBS score (Mean = 40.9) overall: *t* = 4.037, (df = 260.1), *p* < 0.05 (Table 2). Assessing departmental variances there was a significant difference between medicine and surgery (Mean difference = −4.24, *p* < 0.05).

#### 3.1.2 Clinical demands

To ensure scoring was equitable across all questions, the clinical demand questions in the survey were reversed before analysis took place, where the value 1 represents high clinical demand and 5, low demand. Clinical demands questions were combined to give an overall clinical demand score. A mean clinical demand score of 2.6 was found. Perceived clinical demand was lower for males (Mean = 2.76) in comparison to females (Mean = 2.43) *t* = 3.28, (df = 269.8), *p* < 0.05 (Table 3). Analysis of departmental variance showed a significant difference between medicine (Mean = 2.33) and surgery (Mean = 2.83) of perceived clinical demands (Mean difference = 0.501, *p* < 0.05) (Table 3).

**TABLE 3:** Independent *T*‐Test and Analysis of Variance (ANOVA) analyses of clinical demand

Clinical demand score				
NHS scotland (*n* = 131)	NHS England (*n* = 142)		*i t*‐test	
Mean (SD)	Mean (SD)	*t*	df	*p*
2.34 (0.72)	2.86 (0.88)	−5.33	271.0	<0.05

Male (*n* = 145)	Female (*n* = 128)		*i t*‐test	
Mean (SD)	Mean (SD)	*t*	df	*p*
2.76 (0.90)	2.43 (0.74)	3.28	269.8	<0.05

Medicine (*n* = 92)	Surgery (*n* = 86)		ANOVA	
Mean	Mean	Mean difference	SE	*p*
2.33	2.83	0.501	0.121	<0.05

*Note*: Range: 1 (High)—5 (Low).

#### 3.1.3 Managerial support

The managerial support questions were combined to give an overall managerial support score. Descriptive analysis of this dependent variable showed that overall mean managerial support was 2.38 (SD 0.78). The scale for this variable was defined as negative to positive from 1 to 5 with regards to support. Perception of managerial support was significantly lower in NHS Scotland (Mean = 2.12) in comparison to NHS England (Mean = 2.57): *t* = −4.407, (*df* = 258.2), *p*=<0.05 (Table 4). Likewise, perception of managerial support was lower in females (Mean = 2.22) versus males (Mean = 2.52) through independent *t*‐test analysis: *t* = 3.254, (*df* = 270.98), *p* < 0.05 (Table 4). General Practice had a significant difference in comparison to Medicine (Mean difference = 0.446, *p* < 0.05).

**TABLE 4:** Independent *T*‐test and Analysis of Variance (ANOVA) analyses of managerial support

Managerial support score				
NHS scotland (*n* = 131)	NHS England (*n* = 142)	*i t*‐test		
Mean (SD)	Mean (SD)	*t*	df	*p*
2.18 (0.63)	2.57 (0.85)	−4.35	271.0	<0.05

Male (*n* = 145)	Female (*n* = 128)	*i t*‐test		
Mean (SD)	Mean (SD)	*t*	df	*p*
2.52 (0.81)	2.22 (0.71)	3.23	271.0	<0.05

Medicine (*n* = 92)	General practice (*n* = 53)	ANOVA		
Mean	Mean	Mean difference	SE	*p*
2.14	2.59	−0.446	0.13	<0.05

*Note*: Range: 1 (Low)—5 (High).

**FIGURE 3:**
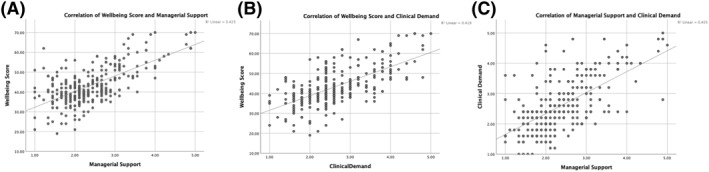
Pearson Correlations—Managerial Support, Clinical Demand, Wellbeing Score. (Scatter plots showing correlations: (A) Managerial support and psychological wellbeing, *r*=0.65, (B) Clinical demand and psychological wellbeing, *r* = 0.647, (C) Managerial support and clinical demand, *r* = 0.636).

To investigate relationships, analysis was conducted using a Pearson's correlation coefficient (*r*) (Figure 3) and linear regression analysis (Table 5).

**TABLE 5:** Linear regression analyses of demographics, clinical demand and managerial support ‐ impact on Warwick‐Edinburgh Mental Wellbeing Scale (WEMWBS) score

Outcome Measure	Significantly related factors	Coefficient estimates	*t*	*p*	*R* ^2^	Adjusted *R* ^2^
WEMWBS score	Clinical demand	4.40	7.15	<0.05	0.52	0.51
	Managerial support	4.87	7.28	<0.05		
WEMWBS score	Gender (1‐Male, 2‐Female)	−3.37	−3.06	<0.05	0.13	0.12
	Age	0.11	2.39	<0.05		
	Region (1‐Scotland, 2‐England)	3.82	3.55	<0.05		
WEMWBS score	Q9.3 ‘I can talk to management about something that has upset or annoyed me’	1.39	2.44	<0.05	0.44	0.43
	Q9.5 ‘When changes are made at work by management, I am clear how they will work out in practice’	2.52	4.33	<0.05		
	Q9.7 ‘Management is willing to invest money and effort to improve safety’	1.65	2.86	<0.05		
	Q9.9 ‘Employee safety practices seem important to management’	2.06	3.94	<0.05		

Linear regression analyses (Table 5) investigated influential factors on wellbeing score. Clinical demand and managerial support demonstrated good fit and accounted for 51% of variance of wellbeing score. Gender, age and region showed significant influence on wellbeing score and accounted for 12% of variance. NHS England as a region and higher age had a positive influence on wellbeing, whilst being female had a significant negative influence.

Strongest correlation (*r* = 0.65) was between managerial support and wellbeing scores. This is suggestive that the higher the managerial support the higher the wellbeing score within the study population. Equally, there is significant correlation between clinical demands and wellbeing score that is, when the clinical demand is high the wellbeing score is lower (*r* = 0.647). A significant relationship also exists between perceived managerial support and clinical demands (*r* = 0.636), where an increase in perception of managerial support results in lower clinical demands within the study population.

#### 3.1.4 Managerial interventions

Regression analysis of specific managerial survey questions against wellbeing score was conducted to investigate the impact of specific management interventions, in line with the study objectives. Table 5 illustrates that the following questions: Q9‐3 ‘I can talk to management about something that upsets or annoys me’; Q9‐5 ‘When changes are made at work, I am clear how they will work out in practice’; Q9‐7 ‘Management is willing to invest money and effort to improve safety’ and Q9‐9 ‘Employee safety practices seem important to management’ demonstrated good fit and accounted for 43% of variance. These categories, based on the statistically significant relationships, are suggestive focus areas which correlate strongly with greater wellbeing score compared to other areas of management.

### 3.2 Interviews

**FIGURE 4:**
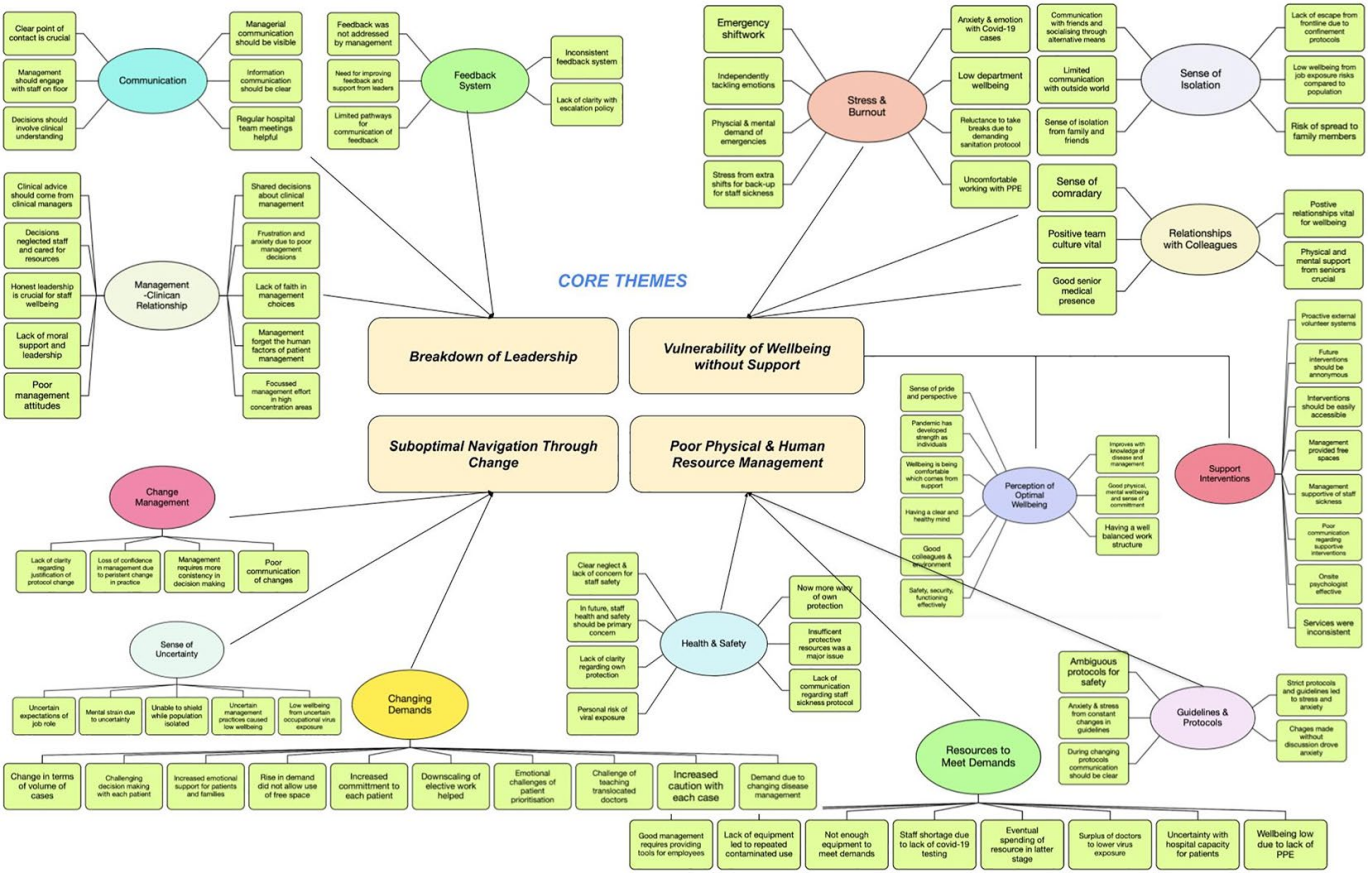
Thematic mapping—development of subthemes and core themes

Semi‐structured interviews were transcribed and coded, and codes were categorised into similar concepts to create subthemes. Demographics of interviewees were two males and two females with grades ranging from foundation to consultant. Two interviewees were from medical specialties, one from surgery and one from general practice. Through further thematic analysis, subthemes were further grouped into more focussed concepts (Figure 4) to create core themes:

**Breakdown of leadership**—Subthemes of ‘communication’, ‘feedback system’ and ‘management‐clinician relationship’ were felt to show similarity, since communication was sporadic with no continuity and decision making from leaders lacked clarity and honesty, making relations vulnerable.
**Vulnerability of wellbeing without** **support**—‘stress and burnout’, ‘sense of isolation’, ‘relationships with colleagues’, ‘support interventions’, and ‘perceptions of optimal wellbeing’, were subthemes based on the perspective of doctors' emotions. These illustrated the toll of the pressures of the work environment, suboptimal support interventions, the lack of escape and how they compensated for this through comradery.
**Poor physical and human resource management**—‘guidelines and protocols’, ‘resources to meet demands’ and ‘health and safety’, were subthemes with common concepts around imbalance of resources, particularly PPE and staffing. This compromised safety and erratic guidelines contributed to this significantly.
**Suboptimal navigation through change**—‘changing demands’, ‘change management’ and ‘sense of uncertainty’ were felt to be closely related subthemes, since the rise in demands were not met with effective change navigation by managers and strongly influenced the perception of uncertainty felt by doctors.

## 4 DISCUSSION

### 4.1 Psychological wellbeing

The findings of the survey showed that the overall mean WEMWBS score was lower (43.2) in comparison to a population mean wellbeing score of 51.6 (SD = 8.7) according to the Health Survey for England 2011.^31^ This supports the literature investigating psychological wellbeing in previous pandemics such as SARS, Ebola and H1N1, which evidenced concerns of low psychological wellbeing of doctors working on the frontline.^22^, ^32^, ^33^ Likewise, this study is in keeping with other similar studies during Covid‐19 which have shown high prevalence rates of poor wellbeing.^34^

The results showed that WEMWBS score was lower for NHS Scotland respondents (Mean = 40.91) in comparison to NHS England (Mean = 45.39), and results were statistically significant. There was also a statistically significant difference between male WEMWBS score (Mean = 45.3) and female WEMWBS score (Mean = 40.9) overall (Table 2). Interestingly, although NHS Scotland had a lower mean wellbeing score in comparison to NHS England, the total number of confirmed Covid‐19 cases in England were 279K cases in comparison to Scotland, which was 21.5K cases at the time if study.^35^ It would have been expected that the NHS region with the largest impact of cases would have had the greatest impact on psychological wellbeing, although this is also dependent on resource and population distribution. This is reflected in a multicentre study of various Asian‐Pacific countries which showed that psychological impact was not dependent on caseload, with Singapore recording the highest volume of Covid‐19 cases but comparatively less psychological impact.^36^ Looking at the demographics of each region, there was a greater percentage of females in the study group of NHS Scotland in comparison to NHS England (Table 1) and there was a lower mean age. Recent studies have shown that being young with less experience, being female and working on the frontline during Covid‐19, were all independent predictive factors of low psychological wellbeing.^5^, ^37^ Our study's regression analysis indicated that being female and lower age had a significant negative influence on psychological wellbeing, equally supporting the literature. In view of this, it may be that demographic profile could have influenced the findings of regional psychological wellbeing scores. Other studies have noted other independent risk factors for adverse psychological outcomes such as individuals who are non‐medical, those with prior medical conditions and presence of physical symptoms.^38^

In terms of departmental variances, there was a significant difference between medicine and surgery wellbeing scores (Mean difference = −4.24, *p* < 0.05). Early studies have observed psychological distress amongst frontline healthcare workers, in particular those in first responder specialties such as medicine, due to high concentration workload.^25^ Two interview respondents were from the frontline medical fields and were able to provide further insight into the concentration of workload:

Respondent 1 “I think my department was the one that was one of the most impacted departments in the hospital… This is acute medicine, is it? Yeah, it was the acute medicine department and the moral was a bit low initially because there was just no consensus of how to manage the situation… we were just blinded by the fire so to speak!”.

Respondent 4 “Our department was medicine and had the highest concentration of cases, so we were even more so on edge and wary about risk of spread”.

Both respondents describe the challenge of increased department workload, but the main driver, which was categorised as a subtheme, was sense of uncertainty (Figure 4). This uncertainty of clinical management and fear of viral transmission associated with increased demand and concentration of cases has also been described in a recent study, and the effects on wellbeing are significant.^37^

### 4.2 Clinical demands

An outcome of the study was to establish the level of clinical demands experienced by doctors during the Covid‐19 pandemic. The results of the survey showed that the overall perceived clinical demand score was 2.6, that is, high demand. This would support the evidence in the literature, which, understandably, has shown that frontline workers face a surge in work demands during global disasters and significant events.^39^, ^40^, ^41^

Other key findings from the survey results were that perceived clinical demand was lower for males (Mean = 2.76) in comparison to females (Mean = 2.43), with a significant difference in demand score (Table 3). With respect to region, NHS Scotland had a statistically significant higher mean combined clinical demand score (Mean:2.34) in comparison to NHS England (Mean = 2.86) (Table 3). As discussed earlier, medical specialties were faced with higher concentration of cases which respondents believed strongly impacted their wellbeing. This correlates with the survey analysis of clinical demand, where medicine experienced higher perceived demand (Mean = 2.32) compared with surgery (Mean = 2,83) as a department (Section 4.2.3). Interestingly, when examining the survey data, 37.4% of responses were from the specialty of medicine in NHS Scotland in comparison to 30.3% in the NHS England group, which may account for the comparative rise in demand in the Scottish study population (Table 1). With regards to difference in gender, there was also a higher percentage of females in comparison to males in the NHS Scotland group (Table 1). Female gender was a significant factor accounting for variance of psychological wellbeing (Table 5). Similar findings were reported in a recent Chinese study which demonstrated that frontline female health workers were at increased risk of low psychological wellbeing during the pressures of Covid‐19.^5^

Respondents felt demands had changed from volume to intensity:

Respondent 1 “The demands in terms of on calls became more about being triply cautious with patients. So, in fact, I felt that the demand for me, the onus of responsibility to make sure we don't spread the virus was more important than the number of patients seen”.

Respondent 3 “The one positive was that we were able to minimise our exposure by downscaling to providing an emergency service. Unfortunately, because of the structure, which was created for the pandemic, our junior doctors were transferred to the medical frontline, which was understandable, however, this meant we would cover two tiers of workload doubling our clinical demand”.

Respondent 4 “So, I wouldn't say the volume was a factor, it was the demand of the extra time, thinking and protocols associated with each patient case. Also, there was so much emotion added to our clinical work. We had the challenges of the department and colleagues who were frustrated, and the emotions of the families of patients to manage. We had to prioritise our patients needs and our personal needs”.

The above comments expand on the experiences of change in demands and it can be noted that, although the volume of patients was counteracted by cancelling elective workload, each patient case was associated with risk of virus exposure and uncertainty requiring more time, protocols and caution amounting to high clinical demand. Although Respondent 3 was not looking after medical patients, the translocation of his junior doctors to the medical team placed a higher workload for his surgical practice. The insights gained from these experiences provide a better understanding of the reasons for rise in demand. Although limited in study, there are recent studies which have also identified the vulnerability of doctors on the frontline including an American study which found a high prevalence of stress and burnout amongst doctors managing Covid‐19 patients.^42^

### 4.3 Managerial support

As explained in Section 1, managerial support is a multidimensional concept and adequate support is based on companionate, informational, practical and emotional terms.^43^ Questions taken from the HSE‐MS IT facilitated identification of characteristics including demands, resources and support within this study.^18^ Survey analysis showed that overall mean managerial support was 2.38 (SD 0.78) for the study population that is, low perception of support. This finding is suggestive that doctors were poorly supported during their experiences of the pandemic and, given that strongest correlation (*r* = 0.65) was found between perceived managerial support and wellbeing scores, this will have influenced overall low psychological wellbeing. Studies, including a systematic review, support the results of this study reporting low managerial support and its negative influence on psychological wellbeing.^41^, ^44^, ^45^, ^46^

There was also a strong correlation between clinical demand and perceived managerial support suggesting that increased managerial support results in significant variance and lower clinical demand (Table 5). Interestingly, perception of managerial support was significantly lower in NHS Scotland (Mean = 2.12) in comparison to NHS England (Mean = 2.57), and as discussed in Section 5.3, clinical demands were much higher in NHS Scotland. In view of this, these relationships can potentially explain the resultant regional outcomes.

### 4.4 Managerial impact on psychological wellbeing

In order to further evaluate the impact of managerial support on the psychological wellbeing of doctors during the pandemic, theoretical models and the emergent theories of the thematic analysis will be drawn upon.

#### 4.4.1 JD‐R model

In Section 2.3, the JD‐R model was discussed as a theory where job demands, physical or psychological, can negatively influence resources and result in negative wellbeing, whilst job resources, psychological, social or physical, can buffer the effects of job demands and also increase motivation and wellbeing.^47^ Evaluating the results of clinical demands, Section 5.3, an increase of demands, physical and psychological as described by respondents, can be applied to this model. The impact of support for resources to mitigate this rise in demand was clearly an issue since, according to the theory, support for resource would buffer and avoid negative psychological wellbeing, which was not the experience of the study population.

#### 4.4.2 Core themes

##### Breakdown of Leadership

Respondents during interviews voiced their concerns regarding inconsistency with feedback systems, poor communication and a breakdown in the management‐clinician relationship:

Respondent 2 “The key here is communication. The NHS works on rationing of services and resources. We all understand as the medical workforce that we may not have everything for everybody but I think you should have somebody at the top who is your point of contact, central to your department who will communicate with you on a daily basis”.

Respondent 3 “Management should take the lead on implementing support personnel to approach and speak to healthcare workers and address any issues. A lot of the time it's more emotional and psychological support face to face…… when we raised concerns these responses were met with no clear solutions and we were given protocol and guidance which was not appropriate”.

Respondent 4 “So overall support should have come in the form of not only continuous communication, but honest communication. Doctors would have been more accepting of change if they were able to understand the rationale and logic behind these choices”.

These responses indicate that there was poor communication from managers at multiple levels and the staff did not feel as if they were heard. Lack of clear planning in terms of communication and escalation pathways resulted in a call for visible leadership. In crisis situations, where effective leadership and decision making becomes paramount, the staff did not feel as if they could trust the managerial team.^3^

##### Suboptimal Navigation through Change

Respondents revealed distress with poor change management leading to uncertainty in the face of rising demands:

Respondent 3 “The persistent changes on a day to day basis clearly impacted how confident we felt with not only our department but also our management system as a whole. I think as a staff member, constant changes make you very uneasy and you can lose confidence as a team”.

Respondent 2 “Uncertainty of a pandemic is inevitable but, this is where leadership should not involve continuous changes of guidance and position. Managers should adapt based on every level including safety not just supply and demand of resource”.

The literature notes that managers ability to withstand disturbance, carry out change whilst retaining functionality is a form of measuring resilience.^10^ Unfortunately, there was continuous change to guidelines and protocols which led to added uncertainty to an already uncertain and high‐pressure crisis.

##### Poor Physical & Human Resource Management

The JD‐R model involves the ideology of physical resource to meet the demands during times of change.^47^ An area of immense priority during a public health pandemic and infectious nature of Covid‐19 would unsurprisingly be PPE and staff safety at work.^48^, ^49^ The nature of this theme is centred on the rise in clinical demand experienced by respondents and the supportive resources and safety in the workplace:

Respondent 1 “So the uncertainty of what we were dealing with and the knowledge that in case the scenario becomes worse for these patients, we won't have the facilities to actually help them at a higher‐level outside of the ward and in the ITU.”

Respondent 2 “It was very difficult at times, for example, once I felt on the ward that I needed more than a surgical mask, I had to argue my case… It was the site manager who we would escalate to in terms of stock and I had to really argue and get my clinical lead involved and had to potentially refuse to see a patient because I felt unsafe!”

Respondent 3 “So unfortunately PPE was a problem from the beginning, and it has always been a problem. I think we were always behind in terms of sufficient support for our safety!”

Respondent 4 “We had limited resources and that was clear from the start so we were in a vulnerable situation.”

The views of respondents suggest significant concern for safety and insufficient resource to meet demands. Lack of resource in a vulnerable situation will have no doubt significantly impacted psychological wellbeing, especially where staff are pleading for safe practice. This reference to PPE supports the literature which has already highlighted the pressure on the NHS from a resource perspective.^3^

##### Vulnerability of Wellbeing without Support

The final theme created from the in‐depth evaluation of the semi‐structured interviews, ‘Vulnerability of Wellbeing without Support’, provides an emotive overview with insights into doctors' vulnerability in terms of isolation, stress, burnout and need for comradery:

Respondent 1 “Unfortunately, normal methods of interactions were not very safe so we were pretty much left to deal with our thoughts and emotions that we were going through on our own”.

Respondent 2 “Wellbeing was quite low and because we were working more hours it was physically more demanding and people were getting more and more tired. There was a lot of fatigue and I think during the whole time there's probably a risk of earlier burnout just because of the sheer number of hours!”

Respondent 3 “In the future senior colleagues have a big role to play to act as role models for junior staff and there should be a positive team culture.”

Respondent 4 “I mean not only was I worrying about my health, I also had to worry about the risk to my family and whether I should isolate myself from them. Also having to isolate from my own family when I was unsure of my viral exposure was emotional”.

The impact of the pandemic in terms of isolation and risk of stress and burnout is apparent in these responses and, as recognised in other studies, the support of managerial teams is paramount to address sources of anxiety and areas of vulnerability.^33^ Effective management strategies include ensuring basic needs of doctors are met, physical health protection and early psychological assessment, monitoring and intervention.^50^ Access to psychological support services is important to buffer the adverse effects of the pandemic as is the identification and removal of any barriers to these interventions.^50^

#### 4.4.3 Summary

The theoretical concept of the JD‐R model and the accounts of the thematic analysis demonstrate how, without resources to meet clinical demands, stress and poor wellbeing is encountered. Evaluation of the emergent themes from the in‐depth analysis of interviews provide key insights into further areas such as lack of navigation of change, support for safety, suboptimal communication which can significantly impact health workers and lead to negative feelings and poor wellbeing.

### 4.5 Limitations

This study has limitations. The cross‐sectional study design, although allowing analysis of multiple variables, is simply a snapshot, whilst the Covid‐19 pandemic is an evolving crisis and experiences of doctors within this environment is dynamic. Additionally, due to feasibility and time, the sample size of 273 survey participants and 4 interviewees is only a fraction of the number of doctors working within the UK health service and therefore limits the scope of the study.

The NHS is an organisation in which each hospital and region have been given capabilities, resources and autonomy to make their own decisions regarding their own institutions. Based on this, to thoroughly assess the full penetration of managerial practices regarding supportive psychological wellbeing approaches across the country and to develop optimal conclusions, it would be necessary to gain abundant data from employees based in every hospital in the country.

Although psychological wellbeing is studied, specific physical symptoms of poor wellbeing amongst doctors was not investigated which could have evaluated in more detail the impact of adverse mental health. Studies have shown differences in psychological impact of Covid‐19 between specialties within surgical and medical departments, although this study only investigated the broad medical and surgical departments which could be seen as a further limitation.^51^

Larger prospective studies are needed to better evaluate the implications of the Covid‐19 pandemic on psychological wellbeing of doctors and the value of managerial interventions.

## 5 CONCLUSION

Doctors are known for their resilience and stamina in the workplace, however the findings highlighted that as learners of a novel virus, as care providers with high virus exposure and being isolated from family and friends amounts to extreme vulnerability and low psychological wellbeing. The findings suggest that with risk of virus exposure and uncertainty of cases, more time, protocols and caution amounted to high clinical pressures. Vulnerable demographics including young, less experienced, female professionals in medical specialties, can influence regional differences in wellbeing. Not only is this compromise of wellbeing concerning, but the uncertainty created by inconsistent managerial guidance, poor feedback systems and failure to maintain an honest path of communication with doctors can generate negative emotion and division.

The study shows that there is a need to formally recognise risk of poor psychological wellbeing amongst healthcare professionals. Equally, educational interventions should be implemented for doctors to allow awareness and use of infection control measures.^52^ The pressures of a pandemic are inevitable, and efforts must be made to ensure demands are met with adequate resources. Resources for psychological support including access to counselling is imperative.^52^

In future crises, it is recommended that basic needs for safety and security should be addressed as a priority. Multifaceted interventions to alleviate isolation and visible leadership with shared decision making is crucial to optimise staff and patient care. Focus should be made on enhancing fundamental managerial principles of control, communication, change management, and leadership through adversity, which are influential targets for maintaining the psychological wellbeing of doctors during crisis. Larger, prospective studies are needed to fully investigate the impact of Covid‐19 on the NHS multidisciplinary workforce, at all levels, and to evaluate in depth adverse psychological wellbeing and how to mitigate this through effective leadership and management interventions.

## CONFLICT OF INTEREST

The authors declare no conflicts of interest.

## ETHICS STATEMENT

The NHS, Invasive or Clinical Research (NICR) ethics panel of the University of Stirling approved the study purpose, design and questionnaires (Decision No: 19/20 – 92, June 2020).

## Data Availability

The data that support the findings of this study are available from the corresponding author upon reasonable request.
